# Vegan/vegetarian diet and human milk donation: An EMBA survey across European milk banks

**DOI:** 10.1111/mcn.13564

**Published:** 2023-09-19

**Authors:** Serena Gandino, Agnieszka Bzikowska‐Jura, Karolina Karcz, Tanya Cassidy, Aleksandra Wesolowska, Barbara Królak‐Olejnik, Daniel Klotz, Sertac Arslanoglu, Jean‐Charles Picaud, Clair‐Yves Boquien, Enrico Bertino, Guido E. Moro, Gillian Weaver

**Affiliations:** ^1^ Nuffield Department of Women's & Reproductive Health University of Oxford, John Radcliffe Hospital Oxford UK; ^2^ Neonatology Division University of Turin, City of Health and Science of Turin Turin Italy; ^3^ Laboratory of Human Milk and Lactation Research at Regional Human Milk Bank in Holy Family Hospital, Department of Medical Biology, Faculty of Health Sciences Medical University of Warsaw Warsaw Poland; ^4^ Department of Neonatology Medical University in Wrocław Wroclaw Poland; ^5^ Kathleen Lonsdale Institute for Health Research Maynooth University Kildare Ireland; ^6^ Department of Neonatology, Center for Pediatrics, Medical Center, Faculty of Medicine University of Freiburg Freiburg Germany; ^7^ Division of Neonatology, İstanbul Medeniyet University School of Medicine İstanbul Turkiye; ^8^ Service de Neonatologie, Hopital Universitaire de la Croix‐Rousse Hospices civils de Lyon Lyon France; ^9^ Laboratoire CarMen, INSERM, INRA Universite Claude Bernard Lyon1 Pierre‐Benite France; ^10^ UMR 1280, PhAN, Nantes Université, INRAE CRNH‐OUEST Nantes France; ^11^ Italian Association of Human Milk Banks (AIBLUD) Milan Italy; ^12^ Human Milk Foundation Rothamsted Institute Harpenden UK

**Keywords:** breast feeding, diet vegan, diet vegetarian, fatty acids, infant nutrition, maternal nutrition, vitamin B12

## Abstract

The nutritional adequacy of human milk (HM) from vegan/vegetarian mothers has been a matter of debate, and a variety of recommendations regarding the eligibility of these mothers as human milk donors exists. According to the latest evidence, HM from vegans/vegetarians is similar in its composition to that from omnivores, however, some differences may be observed regarding vitamin B_12_ and omega‐3 fatty acids concentrations. Maternal supplementation of these compounds has been proven effective in increasing their HM concentration. With this survey, we aimed to explore the practices currently in use in European human milk banks (HMBs) regarding potential donors following vegan/vegetarian diets. The online survey was distributed to European HMBs between January and July 2022. A total of 188 HMBs were contacted, and 118 replied (response rate 63%). Vegan and vegetarian mothers were recommended supplements of vitamin B_12_ to be accepted as donors in 27% and 26% of HMBs, respectively. Additional omega‐3 fatty acid supplementation was required in 8% HMBs. In the remaining HMBs, these mothers were either systematically excluded or included regardless of supplementation. The dosage of the recommended supplements was extremely variable. Fifty‐one percent of HMBs were following recommendations to guide their practice, national or local recommendations mainly. Great variability in European HMBs practices towards potential donors following vegan/vegetarian diets exists. Some of these practices can result in loss of donors and/or in potential nutritional deficiencies. Standardised evidence‐based recommendations on this issue and their implementation in daily HMB practice are needed.

## Key messages

- There is significant variability among the practices of European human milk banks towards donors following a vegan or vegetarian diet.
- The majority of human milk banks follow no guidelines or local guidelines, rather than international ones.
- Some of the practices currently in use can result in the loss of potential donors and/or nutritional deficiencies for mothers, their babies, and potentially for recipients of donor human milk.
- Evidence‐based recommendations regarding the inclusion of these mothers in human milk donation and nutritional counselling are needed.

## Graphical abstract

European Milk Bank Association (EMBA) conducted a survey of European milk banks to explore current practices when recruiting vegan and vegetarian donors. Great variation in practice has been detected. Expert‐based recommendations on this issue are needed. Milk banks can play a crucial health promoting role by providing nutritional counselling to breastfeeding mothers.

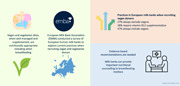

## 1 INTRODUCTION

Extensive evidence demonstrates that human milk (HM) is the optimal nutrition for newborns and infants (Agostoni et al., 2009; Meek & Noble, 2022; Victora et al., 2016). Benefits of HM become particularly crucial in the case of high‐risk infants, such as those born prematurely (American Academy of Paediatrics, 2017; Cassidy & Dykes, 2019; Meek & Noble, 2022). When the mother's own milk is unavailable or insufficient, World Health Organisation (WHO), American Academy of Paediatrics (AAP) and the European Society for Paediatric Gastroenterology, Hepatology and Nutrition (ESPGHAN) recommend donor human milk (DHM) as the preferred feeding choice for preterm infants (Arslanoglu et al., 2013; Meek & Noble, 2022; World Health Organization, 2022). The evidence to support the use of DHM has been widely reviewed (Arslanoglu et al., 2010; Bertino et al., 2013; Landers & Hartmann, 2013; Nolan et al., 2019) especially regarding the reduced risk of necrotising enterocolitis (Campos‐Martinez et al., 2022; Good et al., 2014; Quigley et al., 2019).

DHM composition is related to individual features of donors (e.g., gestational age, maternal diet and nutritional status, stage of lactation and nursing), and to operational procedures (e.g., pasteurisation process, pooling, and mixing practices) established by human milk banks (HMBs) (Bzikowska‐Jura et al., 2021; Italianer et al., 2020). Maternal diet has been recognised as an important variable influencing HM composition, particularly its fatty acid profile and micronutrient content (Ballard & Morrow, 2013; Bravi et al., 2016).

Plant‐based diets have been gaining in popularity in Europe in recent years (Perez‐Cueto et al., 2022), and questions have been raised regarding the adequacy of nutrient intake by vegan and vegetarian women and its impact on HM and DHM composition (Finley et al., 1985b; Perrin et al., 2019). According to the Academy of Nutrition and Dietetics (AND), well‐planned vegan and vegetarian diets are nutritionally appropriate during pregnancy and lactation, for both the mother and her infant (Melina et al., 2016). Due to the reduced content and bioavailability of some nutrients in plant‐derived products, special attention is required for vitamin B_12_, omega‐3 fatty acids, iron, and zinc (Finley et al., 1985a; Ureta‐Velasco et al., 2023). Karcz et al. in their systematic review on vegan/vegetarian diet and HM composition, reported that omnivorous, vegan, and vegetarian mothers produce milk of comparable nutritional value (Karcz & Królak‐Olejnik, 2021). However, lower HM concentrations of specific omega‐3 fatty acids and vitamin B_12_ can be observed in the case of a nonadequately supplemented vegan/vegetarian diet (Karcz & Królak‐Olejnik, 2021). Regular supplementation of these nutrients has been shown to be effective in satisfying maternal needs and translating into their higher concentration in HM, similar to omnivore mothers (Karcz & Królak‐Olejnik, 2021).

Whether the influence of maternal diet on HM composition can affect infant health is a matter of debate, and the evidence is very limited. However, because of the crucial role that DHA and vitamin B12 play in infant neurodevelopment (Cruz‐Rodríguez et al., 2023; Nevins et al., 2021), it seems reasonable to recommend those supplementations to mothers following a vegan/vegetarian diet who are exclusively breastfeeding their babies, and/or donating their milk. Adequate intakes of DHA and vitamin B12 are eventually important for maternal health as well.

According to the European Milk Bank Association (EMBA) recommendations, mothers following a vegan diet should not be excluded from HM donation if they supplement their diet with vitamin B_12_ (Weaver et al., 2019). Other HM banking associations around the world have similar statements, including the Human Milk Bank Association for South Africa (HMBASA, 2014), and the Human Milk Bank Network of Southeast Asia (HMBASA, 2021). Conversely, other national recommendations exclude vegan mothers systematically from HM donation, for example, in Switzerland, Germany, and Poland (Barin & Quack Lotscher, 2018; Richter et al., 2016; The European Foundation for the Care of Newborn Infants [EFCNI], 2018; Wesołowska et al., 2018). Often, these recommendations lack both the definition of vegan diet and the details of the supplementations to be recommended. Therefore, global and evidence‐based guidelines about maternal diet criteria to be included in donor screening, and details of nutritional counselling to be provided to HM donors are still needed.

With this survey, we aimed to explore the role that maternal vegan/vegetarian diet specifically plays in the screening of donors across European HMBs. Our primary objective was to collect information about the policies currently in use in European HMBs for the screening of potential donors following a vegan/vegetarian diet. Our secondary objective was to assess which recommendations, if any, these practices referred to.

## 2 METHODS

### 2.1 Participants

The survey targeted personnel in charge of performing donor screening in European HMBs, but the level of analysis was the HMB, not the individual. Completion was voluntary, and participants were asked to give one answer only from each HMB. In particular, the invitation was sent to the HMBs' managers, who were encouraged in the cover letter to discuss the questions with all the personnel of the HMB.

As the focus of assessment or audit of standard practice within HMB across Europe was on‐going practice and learning, ethical approval was determined not to be needed for this project.

### 2.2 Survey design

The questions were developed and agreed by a team of experts from the EMBA working group ‘Maternal diet and human milk composition’ (Supporting Information: Appendix 1). The survey was tested on Turin (Italy) HMB personnel to check the time required to complete the questionnaire and confirm that the questions were fully comprehended.

The survey comprised of eight closed‐ended questions, three of them asking for open‐ended questions in the case of a positive answer (Supporting Information: Appendix 1). Five to eight minutes on average were necessary to complete the survey.

The survey explored: the practices of European HMBs towards potential donors following a vegan/vegetarian diet; details of supplementations recommended to these donors; adoption of guidelines/recommendations on the issue. Finally, it questioned which other aspects of maternal diet were investigated in donors' screening. In the questionnaire, the term ‘vegetarian diet’ referred to a diet which excluded meat and fish, allowing the consumption of eggs and/or dairy products; ‘vegan diet’ to a diet which excluded the consumption of any animal products: no meat, no fish, no eggs, no dairy products (WHO, 2021). These definitions were pointed out in the original survey at each related question.

### 2.3 Recruitment

An invitation‐only online survey was developed, and this invitation was supplemented with follow‐up phone calls to ensure that the invitation has been received and potentially increase the response rate (Ball, 2019).

Recruitment occurred between January and July 2022. The web‐link was sent via email along with a description of the study rationale and aim. Data were collected through the Survey Monkey tool. The survey was initially sent in English to the whole mailing list, followed by two reminders.

The survey invitation was sent by the EMBA secretariat to all European HMBs included in the EMBA mailing list and performing donor recruitment (first round). Afterwards, the working group researchers were actively involved in the survey delivery management. Three strategies were applied to increase the response rate during the second round. First, an update of EMBA HMBs mailing list was performed and resulted in 25 HMBs being added to the contact list. Second, the survey was translated into French, Italian and Polish. Third, HMBs in Germany, Italy, Poland and UK were invited to complete the survey by an EMBA member active in the HMB network of the specific country.

### 2.4 Data analysis

All returned questionnaires were reviewed separately by two researchers (S. G. and A. B‐.J). Since one reply only was accepted from each HMB, in the case of multiple responses from the same HMB if the answers were identical, one answer only was included in the analysis. If the answers differed, the HMB was contacted via email/phone and asked to provide a definitive answer and explanation for the different replies previously received. Returned questionnaires with no clear HMB identification details were excluded from the analysis.

Data were analysed by using IBM SPSS Statistics for Windows, Version 27 (IBM Corp.). Descriptive data were reported as frequency (percentage) for categorical variables. All calculated percentages were rounded up to the nearest integer. Depending on the distribution of the quantitative variables (the dosages of recommended supplementation), median and inter‐quartile range were used. To test the normality of the numerical data we used Shapiro–Wilk test. Qualitative codes were agreed by two independent researchers specialised in different disciplines (S. G.—neonatology, A. B‐.J.—nutrition) (Coates et al., 2021).

## 3 RESULTS

### 3.1 Sample

**Figure 1:**
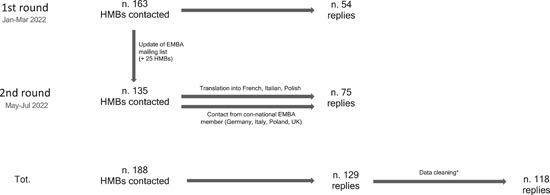
Flow diagram of data collection and data cleaning. *Six replies were excluded because of multiple identical responses from the same HMB; two replies were excluded because of multiple different responses from the same HMBs (the HMB was contacted over phone/email and ask for explanation and a definite answer); three replies were excluded because of unclear HMB identification details.

**Figure 2:**
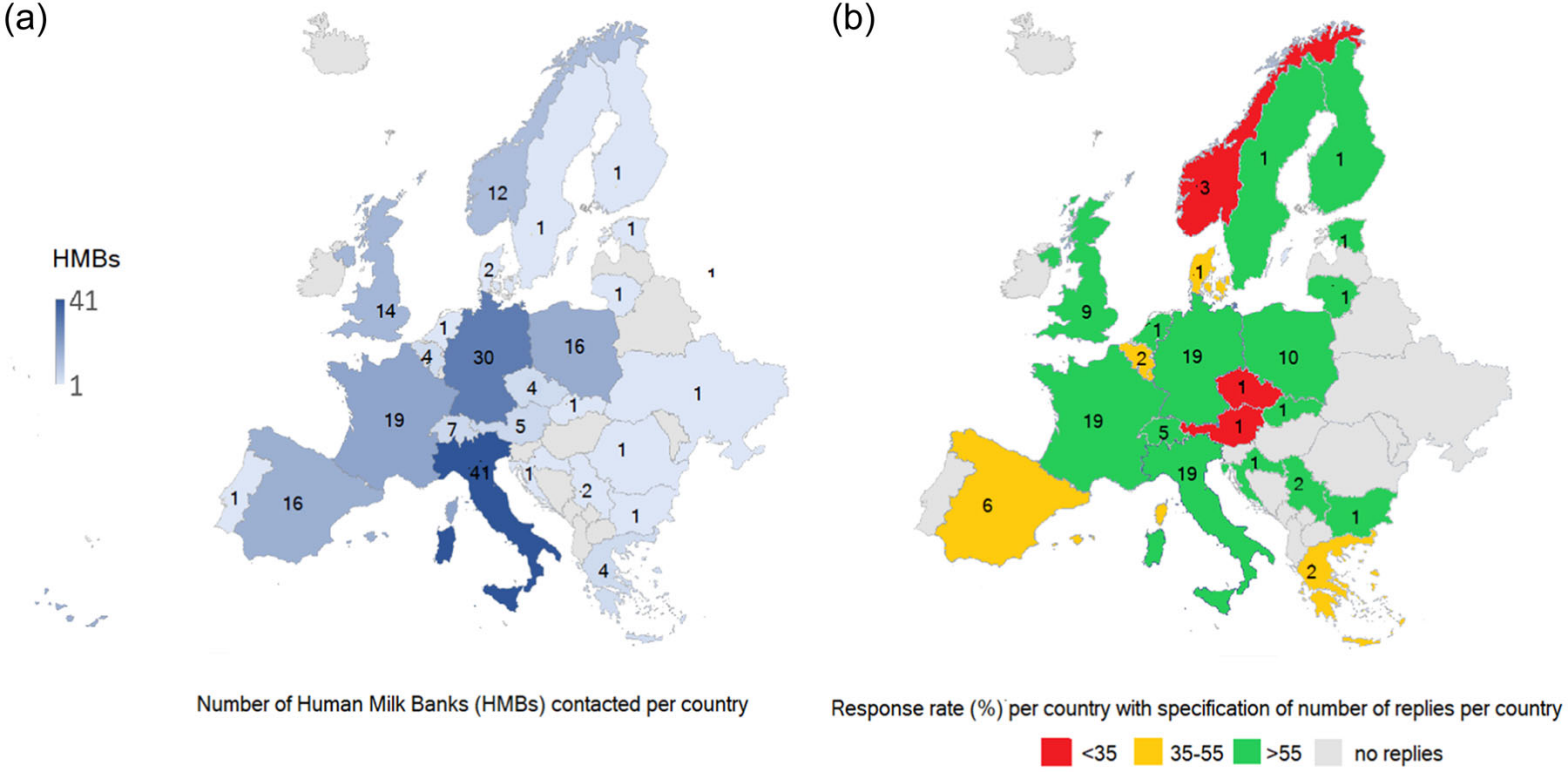
Human milk banks invited to take part in the study (a) and response rate per country (b).

A total of 188 European HMBs in 26 European countries were contacted, and 129 answers were received. Following data cleaning, the response rate was 63% (118/188) (Figure 1). Replies were received from 22 countries. The major contributors (accounting together for 57% of total replies) were Italian, French, and German HMBs (Figure 2). The response rate per each question ranged from 93% (110/118) to 99% (117/118).

### 3.2 Recruitment of vegan/vegetarian donors

Significant variation in exclusion criteria based on vegan/vegetarian diet was seen between milk banks.

As Table 1 shows, when asked if vegan/vegetarian diet was an exclusion criterion to HM donation, just over a quarter of HMBs replied that they always exclude vegan donors whereas only six percent said that they always exclude vegetarian donors (Table 1). Donation was allowed when vitamin B_12_ was supplemented in a quarter of HMB if the mother was vegan or vegetarian. Vegan mothers were always included in HM donation, regardless of vitamin B_12_ status and supplementation in 40% of HMBs, vegetarians in 64%.

**Table 1:** Results from questions investigating the practise of HMBs related to vegan/vegetarian potential donors.

In your HMB do you exclude a potential donor if she follows a…	Vegan diet, *n* (%)	Vegetarian diet, *n* (%)
Yes, always	32 (27)	7 (6)
No, if the mother supplements her diet with vitamin B_12_	23 (20)	21 (18)
No, if the mother supplements her diet with vitamin B_12_ and omega‐3 fatty acids	9 (8)	9 (8)
No, if biochemical parameters are judged to be normal (mainly B12)	1 (1)	2 (2)
Never/we don't ask for this information	47 (40)	76 (64)
No standardised approach	4 (3)	1 (1)
Other^a^	1 (1)	1 (1)

^a^ One HMB was accepting vegan and vegetarian women as donors if they were taking undefined nutritional supplements, without investigating further the supplementation.

### 3.3 Recommendations followed

To guide the inclusion of vegan/vegetarian mothers in HM donation, 53/113 HMBs (47%) were not following any recommendation; 40/113 (35%) were following national recommendations, 14/113 (12%) local recommendations. EMBA recommendations were followed by 5/113 HMBs only (4%), other European recommendations (‘Guide to the quality and safety of tissues and cells for human application’ in 1/113 HMB (1%).

### 3.4 Vitamin B_12_ status and supplementation

The vast majority of HMBs (108/117, 92%) did not check maternal blood levels of vitamin B_12_ at the donor screening. Vitamin B_12_ levels were investigated in 6/117 (5%) HMBs if the mother was vegan, in 3/117 (3%) if vegetarian.

Most HMBs (68/110, 62%) did not recommend vitamin B_12_ supplementation. Among HMBs that recommended vitamin B_12_ supplementation, 24/42 (57%) have defined the specific dose of the supplementation, 5/42 (12%) reported ranges (e.g., 4.5–5.5 µg/day), remaining 21/42 (50%) did not specify the dosage. Considering the specific doses of vitamin B_12_ supplementation, the median recommended value was 5.5 [0.5; 571.4] µg/day.

### 3.5 Omega‐3 fatty acids supplementation

Omega‐3 fatty acids supplementation was recommended in 25/114 (22%) HMBs. 12/25 HMBs specified the exact dosage of the supplementation, while in two cases the dosages were presented as ranges. The median recommended value was 200 [150; 600] mg/day. Two HMBs specified that the supplementation was for docosahexaenoic acid (DHA) only.

Omega‐3 supplementation was a conditional requirement to become a donor in case of vegan/vegetarian diet in 8% (9/117) HMBs.

### 3.6 Maternal diet and HM donation

**Figure 3:**
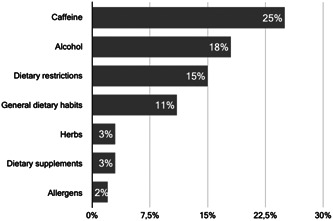
Proportion of European HMBs (*n* = 114) which investigated other aspects of maternal diet during the donors' screening.

A total of 62/114 (54%) HMBs did not investigate further aspects of maternal diet during the donor screening. Those who did (52/114; 46%) were asked to specify which other aspects of maternal diet were included in the donors' screening in their HMBs. Replies were analysed by qualitative coding (Braun & Clarke, 2022). The largest attention was found to be paid to alcohol and caffeine intake (Figure 3).

## 4 DISCUSSION

This study provides insights into the practices of European HMBs towards potential donors following a vegan or vegetarian diet. We found an extreme variation in the practices currently in use to screen donors' diet in European HMBs, both among different countries as well as within the same country.

According to EMBA recommendations (Weaver et al., 2019), maternal diet should be investigated during the donors' screening, and vegan mothers included in HM donation if vitamin B_12_ is adequately supplemented. The rationale of this recommendation is the well‐known role of maternal diet in influencing certain HM components (Bravi et al., 2016), and the risk of vitamin B_12_ deficiency associated with plant‐based diets, when not adequately planned and supplemented. Vitamin B_12_ is crucial for infant neurodevelopment and its concentration in HM relates to maternal stores and blood levels (Specker et al., 1990).

In our study, we found that only one quarter of European HMBs required vitamin B12 supplementation to vegan/vegetarian mothers to be included as donors. These banks followed a similar practice towards vegan and vegetarian mothers. The risk of vitamin B_12_ deficiency may indeed be comparable in these two diets since vitamin B_12_ is a water‐soluble vitamin, whose natural sources are all animal‐derived. Theoretically, the more restrictive the plant‐based diet is, the higher the risk of nutritional deficiency (Niklewicz et al., 2022). Practically, many vegetarians cannot cover vitamin B_12_ needs with the number of animal‐derived foods (eggs and dairy products) that they consume. Conversely, vegans tend to more frequently consume fortified foods (foods containing vitamins and minerals that are not naturally present in the diet, added to prevent micronutrients deficiencies). The risk of vitamin B_12_ deficiency is, therefore, similar among these two groups (Baroni et al., 2018; Niklewicz et al., 2022; Sebastiani et al., 2019).

From our survey, one HMB out of four systematically excluded vegan mothers from HM donation. This may be due to the warnings that several scientific associations, for example, the ESPHGAN, and the German Nutrition Society, recently made regarding the risk of nutritional deficiencies associated with plant‐based diets (Fewtrell et al., 2017; Richter et al., 2016). Nevertheless, the AND underlined that plant‐based diets, when adequately planned, can provide appropriate nutrition during pregnancy and lactation (Melina et al., 2016). To be considered as ‘adequately planned’, the plant‐based diet must comprise a wide variety of plant foods, mainly unprocessed; vegetable fats must be chosen carefully, by limiting monounsaturated and polyunsaturated omega‐6 fatty acids, and by preferring omega‐3 fatty acids, adequate intakes of calcium‐rich plant foods should be consumed, and vitamin B_12_ must be supplemented (Baroni et al., 2018; Fewtrell et al., 2017; Melina et al., 2016; Sebastiani et al., 2019; Van Winckel et al., 2011). If these criteria are respected, vegan and vegetarian diets are nutritionally adequate and can be considered healthy. Excluding vegan mothers who meet these criteria from HM donation would just result in losing potential donors.

On the other hand, from our study, it resulted that 40% of HMBs always included vegan mothers in HM donation, regardless of vitamin B_12_ supplementation. This may partly be due to an underestimation of the proportion of childbearing women following exclusive plant‐based diets. However, the adherence to vegan/vegetarian diets in Europe has been increasing steeply over recent years because of ethical, religious, health, environmental, and economic reasons, and it is expected to rise further in the next decade (Perez‐Cueto et al., 2022). Women in particular are more prone to adopt plant based‐diets (Satija et al., 2019). Therefore, it is very important to investigate this dietary choice during the donor screening and have clear recommendations for the nutritional counselling of these donors. As previously mentioned, plant‐based diets (when not fortified or supplemented) may lead to vitamin B_12_ deficiency (Melina et al., 2016; The European Foundation for the Care of Newborn Infants EFCNI, 2018). Data regarding the impact of a maternal plant‐based diet on the vitamin B12 concentration of HM remain inconclusive, whereas the use of appropiate supplements appear to compensate for any dietary effects (Karcz & Królak‐Olejnik, 2021). Extensive evidence has shown that vitamin B_12_ deficiency in infants can lead to severe clinical consequences, particularly neurodevelopmental impairments (Baroni et al., 2018; Fewtrell et al., 2017; Richter et al., 2016; Sebastiani et al., 2019; Van Winckel et al., 2011). Mother's vitamin B_12_ supplementation has been proven to be effective in increasing HM vitamin B_12_ content (Baroni et al., 2018; Karcz & Królak‐Olejnik, 2021; Pawlak et al., 2018; Sebastiani et al., 2019). To reach adequate vitamin B_12_ levels, it is recommended that the mother takes an individual B_12_ supplement, not relying on her vitamin B_12_ intake on multivitamin supplements or fortified foods alone (Pawlak et al., 2018). Holder pasteurisation of DHM (commonly used in HMBs), does not significantly affect HM vitamin B_12_ content (Van Zoeren‐Grobben et al., 1987). Therefore, individual vitamin B_12_ supplementation in donors following a vegan/vegetarian diet may be an effective strategy to ensure adequate vitamin B_12_ levels in DHM.

As outlined by Karcz et al., HM from vegan and vegetarian mothers may also differ from HM from omnivorous mothers in the profile of fatty acids (Karcz & Królak‐Olejnik, 2021). Perrin et al. found significantly higher concentration of alpha‐linolenic acid (ALA) in vegan mothers (2.09%) in comparison to vegetarian (1.55%) and omnivores mothers (1.19%) (Perrin et al., 2019). Interestingly, the authors did not find significant difference in DHA content in any of three dietary groups. Additionally, the use of DHA and/or EPA supplements was a significant positive predictor total omega‐3 FAs content in HM and a significant negative predictor of omega‐6: omega‐3 ratio. Contrary to these findings, in the latest Spanish study (Ureta‐Velasco et al., 2023) it was observed that HM DHA content in omnivores donor mothers was double than in mothers following a vegetarian diet (0.33 vs. 0.15/100 g of total fat). DHA and EPA are long‐chain omega‐3 fatty acids abundant in fish, shellfish and seaweed, and they play a crucial role in the development of the brain, retina, and cell membranes. EPA and DHA can be endogenously synthesised from alfa‐linolenic acid (ALA), whose intakes and levels are similar among vegetarians and omnivores (Melina et al., 2016). However, this endogenous conversion process is highly inefficient and cannot cover the increased DHA requirements that occur during pregnancy and lactation (Baroni et al., 2018; Melina et al., 2016; Sebastiani et al., 2019). It is therefore recommended that pregnant and breastfeeding women who do not consume fish, should supplement 100–200 mg of DHA daily (Baroni et al., 2018). In our study, only nine HMBs (8%) required omega‐3 fatty acids supplementation in addition to vitamin B_12_ supplementation to vegan and vegetarian donors. Pasteurisation of HM preserves omega‐3 fatty acids (Fidler et al., 2001; Moltó‐Puigmartí et al., 2011), therefore, fatty acids omega‐3 supplementation in donors who do not consume fish may be an effective strategy to improve the nutritional quality of their milk.

Only a minor proportion of the HMBs which recommended vitamin B_12_ and/or omega‐3 fatty acids supplementation specified the dosage of these supplements. Among those who did specify it, a great variation in the dosage was detected. This data outlines the need for clear and shared recommendations on this topic.

From our study, it resulted in most European HMBs were following no or local/national recommendations to guide their practice. Analysis of replies regarding current practices confirmed a tendency to follow national recommendations. In Germany and Poland, more than 55% of HMBs systematically excluded vegan donors. In Italy, vegan mothers were included in HM donation if the diet was supplemented with B_12_ in 52% of cases, in line with HMB Italian Association (AIBLUD) recommendations. In France, where there are no recommendations on this topic, 74% of HMBs did not investigate whether the mother is vegan or vegetarian at the donor screening. In the United Kingdom, where eight out of nine HMBs replied that they were not following recommendations on the topic, the practice regarding vegan/vegetarian donors was extremely variable.

Given the role of maternal diet in influencing some HM components and given the potential harmful effects of specific substances (e.g., alcohol, caffeine) when present in high concentrations in HM (EFSA, 2015; Haastrup et al., 2014; NHS, 2022; NIH, 2006; Temple et al., 2017; Wilson et al., 2017), these further aspects of maternal diet require proper investigation during the donor screening (Weaver et al., 2019). However, our survey results showed that less than half (52/114; 45%) of European HMBs were investigating these aspects at the donor screening with caffeine and alcohol intake being questioned only in 25% (29/114) and 18% (20/114) cases, respectively. This very low tendency to investigate such important issues might be explained by cultural variations as well as a lack of strong evidence and recommendations on the topic. In particular, evidence on how the screening for these lifestyle behaviours affects human milk composition is lacking. More research is needed to elucidate these aspects of donors' screening.

### 4.1 Limitations

Although most replies to this survey were received from a limited number of countries, they represented two‐thirds of existing European HMBs, and we do not have strong reasons to postulate a different pattern of practices in the HMBs that did not reply.

A further limitation is the reliance on data provided by respondents: we did not verify if the provided answers were compatible with the practice of the HMB. However, EMBA members were actively involved in supporting the data collection in their countries, monitoring that questions were fully comprehended and adequately answered.

Finally, data on the rates of vegan/vegetarian milk donor‐candidates in each HMB were not available. Therefore, variations in practice were not adjusted for the frequency of consultations given to the mentioned women.

Despite these limitations, the results of the survey provide helpful information to understand baseline practices in different HMBs and in different geographic locations. In particular, the data collected are sufficient to disclose the need for common recommendations on the aspects of maternal diet that should be investigated during the donors' screening and on their management.

## 5 CONCLUSIONS

Our study showed a great variability in the policies that European HMBs apply towards potential donors following vegan/vegetarian diets, and a tendency to follow national or local policies rather than international recommendations. Only a minor proportion of HMBs adopted an ‘evidence‐based’ approach, by including in HM donation mothers following a vegan/vegetarian diet if adequately supplemented. Other approaches, for example, always including vegan donors, always excluding vegan donors, not investigating the donor's diet, can all have detrimental consequences. The main negative effect that can follow any of these practices is missing the chance to recommend proper supplements to the breastfeeding mother, with possible negative consequences for the health of her own baby, who will receive only that milk as a nutritional source for the first 6 months. The inclusion of vegan donors, regardless of any supplementation, can also impair the nutritional quality of DHM. However, this effect may be mitigated by the DHM pooling strategies commonly adopted by some HMBs, and by the common practice of directly supplementing preterm babies with vitamins, either via parenteral nutrition and via enteral supplements once they approach full enteral feeding. Therefore, investigating restricted diets, as well as other aspects of maternal diet, during the donor's screening is primarily important to offer proper nutritional counselling to these mothers, with important benefits to the mother herself, to her own child, and to the babies that will receive her donor milk.

HMBs can play a crucial health‐promotion role by providing adequate nutritional counselling to breastfeeding mothers. However, to do so, HMBs need to be provided with clear instructions on which questions to ask during the donor's recruitment, and on the nutritional counselling to offer, both in general and in specific situations, such as the case of donors following a plant‐based diet.

It is therefore essential to develop common expert‐based recommendations on this issue, and to actively implement these recommendations in the daily practice of European HMBs.

## AUTHOR CONTRIBUTIONS

Serena Gandino, Agnieszka Bzikowska‐Jura, Karolina Karcz, Aleksandra Wesolowska, Barbara Królak‐Olejnik, Enrico Bertino, Guido E. Moro and Gillian Weaver designed the research study. Serena Gandino, Agnieszka Bzikowska‐Jura, Daniel Klotz and Gillian Weaver performed the research. Serena Gandino and Agnieszka Bzikowska‐Jura analysed the data. Serena Gandino, Agnieszka Bzikowska‐Jura, Karolina Karcz and Tanya Cassidy wrote the paper. Aleksandra Wesolowska, Barbara Królak‐Olejnik, Sertac Arslanoglu, Jean‐Charles Picaud, Clair‐Yves Boquien, Enrico Bertino, Guido E. Moro and Gillian Weaver revised the paper.

## CONFLICT OF INTEREST STATEMENT

The authors declare no conflict of interest.

## Supporting information

Supporting information.

## Data Availability

The data that support the findings of this study are available from the corresponding author upon reasonable request.
