# Supplementary material for: Vegan/vegetarian diet and human milk donation: An EMBA survey across European milk banks
Source: Matern Child Nutr. 2023 Sep 19;20(1):e13564. doi: 10.1111/mcn.13564 (PMC10750000; doi:10.1111/mcn.13564)
Supplement: Supplementary file 1 — Supporting information. [file MCN-20-e13564-s001.docx]

Supplementary Appendix 1. Questionnaire delivered to HMBs in the present study.

| Identification of the HMB {country-town-hospital}  1. In your human milk bank, is following a vegetarian diet* an exclusion criterion for human milk donation?  - Yes, always  - No, if the mother supplements her diet with vitamin B12  - No, if the mother supplements her diet with vitamin B12 and omega-3 fatty acids  - Never  - We don’t ask for this information  - Other ___ please specify    *In this survey, vegetarian diet refers to a diet which excludes meat and fish, allowing the consumption of eggs and/or dairy products.    2. In your human milk bank, is following a vegan diet* an exclusion criterion for human milk donation?  - Yes, always  - No, if the mother supplements her diet with vitamin B12  - No, if the mother supplements her diet with vitamin B12 and omega-3 fatty acids  - Never  - We don’t ask for this information  - Other ___ please specify    *In this survey, vegan diet refers to a diet which excludes the consumption of any animal products (no meat, no fish, no eggs, no dairy products; honey and beeswax allowed)    3. Do you check maternal blood levels of vitamin B 12 in the donors’ recruitment screening (performing a blood test during the donor screening or checking previous blood tests performed during the last 6 months)?  - Always  - Only if the mother follows a vegetarian or vegan diet  - Only if the mother follows a vegan diet  - Never  - Other ____please specify    4. In your centre, if you recommend vitamin B 12 supplementation for lactating women, which recommendations are you following for the supplementation?  - Local recommendations --- please specify the dosage of the supplementation  - National recommendations --- please specify the dosage of the supplementation  - European recommendations --- please specify the dosage of the supplementation  - We don’t recommend vitamin B 12 supplementation  - Other __ please specify    5. In your centre, if you recommend omega-3 fatty acids supplementation, which recommendations are you following for the supplementation?  - Local recommendations --- please specify the dosage of the supplementation  - National recommendations --- please specify the dosage of the supplementation  - European recommendations --- please specify the dosage of the supplementation  - We don’t recommend omega-3 fatty acids supplementation  - Other __ please specify    6. In your human milk bank, do you follow any guidelines or recommendations for including/excluding vegan or vegetarian mothers from milk donation?  - Yes, local recommendations  - Yes, national recommendations/guidelines  - Yes, EMBA guidelines  - Yes, other European recommendations/guidelines____please specify  - Yes, others ____please specify  - No    7. In your human milk bank do you ask any other questions related to maternal diet?  - Yes --- please specify  - No    8. Would you be happy to take part in other short EMBA surveys regarding maternal diet in the future?  - Yes  - No |
| --- |
